# Tick Infestation in Migratory Birds of the Vistula River Valley, Poland

**DOI:** 10.3390/ijerph192113781

**Published:** 2022-10-23

**Authors:** Zbigniew Zając, Joanna Kulisz, Renata Kunc-Kozioł, Aneta Woźniak, Maciej Filipiuk, Robert Rudolf, Katarzyna Bartosik, Alejandro Cabezas-Cruz

**Affiliations:** 1 Department of Biology and Parasitology, Medical University of Lublin, Radziwiłłowska 11, 20-080 Lublin, Poland; 2 Department of Zoology and Nature Protection, Institute of Biological Sciences, Maria Curie-Skłodowska University, Akademicka 19, 20-033 Lublin, Poland; 3 Kaliszany Ornithological Station, 24-340 Stare Kaliszany, Poland; 4 Anses, INRAE, Ecole Nationale Vétérinaire d’Alfort, UMR BIPAR, Laboratoire de Santé Animale, F-94700 Maisons-Alfort, France

**Keywords:** ticks, bird, ticks infesting birds

## Abstract

Migratory birds play an important role in the eco-epidemiology of tick-borne diseases due to their ability to carry ticks for long distances. The aim of the present study was to investigate the prevalence and factors influencing the intensity of tick infestation in migratory birds. The study was conducted in a locality situated in the Vistula River valley, eastern Poland, during autumn, when the high migratory activity of birds is registered in the region. The birds were captured using ornithological nets and identified at the species level. In the next step, they were carefully inspected for attached ticks. Tick infestation was observed in 4.43% of the captured birds. The highest mean intensity of tick infestation was observed in birds foraging on the ground or in low shrubs and by long- and medium-distance migrants, i.e., *Turdus merula* (2.73), *T. philomelos* (2.04), and *Erithacus rubecula* (1.58). *Ixodes ricinus* was found to infest the birds most frequently. However, other tick species, i.e., *I.* *trianguliceps*, *I.* *crenulatus* (synonym *I.* *canisuga*), and *I. apronophorus*, rarely found in eastern Poland, were also found parasitizing birds. The occurrence of *I.* *persulcatus*, *I. frontalis*, and *I.* *acuminatus* (synonym *I.* *redikorzevi*) was confirmed in the region for the first time. The results of the study suggest that captured bird species are susceptible to tick infestation and could play an important role in the circulation of some tick-borne pathogens. They also play a significant role in the spread of ticks. The ecology and ethology of birds, including their foraging styles and migratory habits, are factors determining the risk of exposure of birds to tick attacks.

## 1. Introduction

Birds are hosts of a wide variety of parasites representing different taxonomic groups, such as protozoa, helminths, and arthropods [1,2,3]. Given their potential to transport ticks and their role as reservoirs of many pathogenic microorganisms posing a threat to human health, birds play an important role in the eco-epidemiology of infectious diseases transmitted by tick vectors [4,5,6,7,8,9]. In this aspect, the role of migratory birds is particularly important, as they can cross geographical barriers and spread ticks over long distances, even thousands of kilometers [10]. In contrast, ixodid ticks, such as *Ixodes scapularis* Say, 1821, are able to cover a short distance of 2–5 m in natural conditions, depending on the developmental stage [11]. The role of migratory birds in the spread of new alien tick species in Europe, e.g., the *Hyalomma* genus in Germany, has also been confirmed [12]. Similar observations have been reported in Great Britain [13]. In turn, tick species that are endemic to northern Africa were collected, e.g., in Italy, from birds during their seasonal migrations [14].

In Europe, a high infection rate of tick-borne pathogens has been reported in ticks infesting birds [15], with *Borrelia* spirochetes, the causal agent of Lyme disease (LD), being the most frequent [16,17,18]. Other frequently detected pathogens include *Rickettsia* (mainly *R. helvetica*), *Anaplasma phagocytophilum*, *Coxiella burnetii*, *Candidatus* Neoehrlichia mikurensis, *Babesia* spp., tick-borne encephalitis virus (TBEV), and Crimean-Congo hemorrhagic fever virus (CCHFV) [15,19,20,21,22,23,24,25,26]. Co-infections, in particular with various *Borrelia* genospecies [4,15,20], are a frequent phenomenon as well [15,19]. 

In the study area (eastern Poland), *I. ricinus* Linnaeus, 1758 and *Dermacentor reticulatus* Fabricius, 1794 are the most common tick species, and their density is one of the highest in the scale of the whole country [27,28]. Moreover, eastern Poland should be considered an endemic area of tick-borne diseases. A high incidence of tick-borne diseases, especially LD [28], has been reported. Attention is drawn to the high prevalence of tick-borne pathogens detected in ticks; e.g., depending on the habitat, up to 91.7% of adult *D. reticulatus* ticks were positive for *Rickettsia* spp. [29]. Additionally, a high infection rate of *Borrelia burgdorferi*, *Bartonella* spp., *Anaplasma phagocytophilum*, *Candidatus* N. mikurensis, and *Babesia* spp. in ticks collected from vegetation is also observed in the region [29,30]. The presence of *B. burgdorferi* spirochetes was also confirmed in ticks removed from the inhabitants of the region [31]. Although there are no precise data on the role of animals, especially small rodents, in the circulation of tick-borne pathogens in the studied region, previously published results confirmed the occurrence of *Apodemus agrarius* Pallas, 1771; *Microtus arvalis* Pallas, 1778 and *Myodes glareolus* Schreber, 1780 [32], which are important links in enzootic cycles of circulation of tick-borne pathogens in the environment [33,34].

The aim of the present study was to investigate the prevalence and factors influencing the intensity of tick infestation in birds migrating through eastern Poland. To the best of our knowledge, there are no such reports from this region, and only a few studies on the national scale, which is the rationale behind undertaking this research [18,35,36,37,38,39]. Additionally, the location of the study area in the Vistula River valley, which constitutes one of the most important bird migration routes in Poland, is also noteworthy [40].

## 2. Materials and Methods

### 2.1. Ethical Statement

The study design, including bird capture and collecting ticks from them, was approved by the General Directorate of Nature Conservation in Warsaw (DZP-WG.6401.102.2020.TŁ) and the Regional Directorate of Nature Conservation in Lublin (WPN.6401.108.2021.KC).

### 2.2. Study Area

The field study was conducted in cooperation with the Kaliszany Ornithological Station (51°04′45″ N 21°48′04″ E), eastern Poland, which is part of the National Bird Ringing Station Network and the European Bird Migration Network (EBMN). The research station is located on a river island within the Vistula River Gorge of Lesser Poland. The river is a natural bird migration corridor, while the island is a stopover site for migrating birds. The station is situated on the bird migration route between northern and northeastern Europe and the southern and southwestern parts of the continent [41].

### 2.3. Bird Capture Procedure and Tick Collection

The study was carried out during the autumn migration in 2021 over 53 consecutive days, with a 9-day break at the beginning of September due to local flooding.

**Figure 1:**
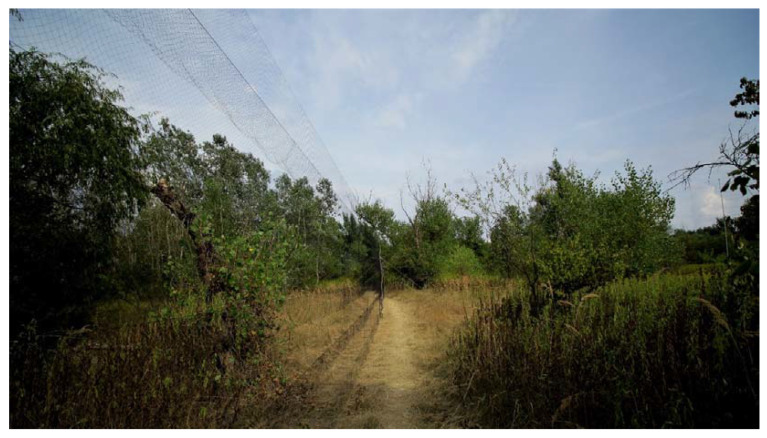
Ornithological net used to catch birds during the study. Photo by R. Rudolf.

The birds were captured in ornithological nets (Ecotone, Gdynia, Poland) with a total length of 500 m, which were stretched along a transect (a total of 1.5 km) running through riverside thickets dominated by riparian vegetation transforming into a meadow community (Figure 1). In principle, the nets were inspected every hour, but these periods were shortened to half an hour in unfavorable weather conditions (rainfall, low or very high air temperatures). The captured birds were carefully released from the net and identified to the species [41,42,43]. Additionally, their sex, age, and amount of fat tissue were determined based on their morphological traits where possible. All birds were weighed using an Ohaus compact portable Ohaus balance (Series CL—CL501) and ringed. These steps were performed in accordance with the protocol proposed by Operation Baltic and EBMN, as described by Zaniewicz and Rosińska [44]. 

Subsequently, the whole birds’ body was carefully inspected for the presence of ticks. Parasites were removed from the body of the birds with tweezers and placed in an Eppendorf tube containing 70% ethanol solution. Samples were transported to the laboratory, where a Zeiss STEMI DV4 stereoscopic microscope (Carl Zeiss Light Microscopy, Göttingen, Germany) and tick identification keys [45,46,47] were used to identify the species, sex, and developmental stage. In the case of tick species that undergo discussion of their species distinctiveness, synonyms were applied [46,47,48]. When the identification of the tick species was impossible due to damage to the body, the specimens were identified at the genus level. Due to the identification of tick species only on the basis of morphological characteristics, the features typical for the identified species and developmental stages are summarized in Supplementary Tables S1 and S2.

### 2.4. Analysis of the Quantitative Structure of Tick Populations and Their Host Preferences

The quantitative structure of the tick population was analyzed by calculating the ecological index of dominance (DTs%) and the index of prevalence (PTs%) using the following formulas [49,50]:

$$DTs=\frac{Ts}{Tt}\times 100\%$$

where *DTs*—ecological index of tick dominance, *Ts*—number of ticks of a given species collected from all birds, *Tt*—total number of ticks collected from all birds.

$$PTs=\frac{BTs}{Bt}\times 100\%$$

where *PTs*—prevalence of particular tick species, *BTs*—number of birds infested by a particular tick species, Bt—total number of examined birds.

The host preferences of the analyzed tick populations were determined based on an indicator called the preference index according to the formula proposed by Dudich [51]. Due to the possibility of overestimating the result for calculations with a small sample, only bird species with at least 100 captured individuals were included in the analysis:

$$I_{p}=\frac{TsBs\times Bt}{Ts\times Bs}$$

where $I_{p}$—preference index, *TsBs*—number of ticks of a given species collected from a given bird species, *Bt*—total number of examined birds, *Ts*—number of ticks of a given species, *Bs*—number of birds of given species, *I_p_* < 1—negative interaction; *I_p_* = 1—neutral interaction; *I_p_* > 1—positive interaction. 

Additionally, the number of host species infested by a particular tick species (NHSPI) (Baláž and Zigová) [52] and the mean intensity of infestation (*MII*) of the birds by a particular tick species were calculated as in Bush et al. [53]:

$$MII=\frac{Ts}{BTs}$$

where *MII*—mean intensity of infestation of particular tick species; *Ts*—number of ticks of a given species; *BTs*—number of birds infested by a particular tick species.

#### 2.4.1. Ornithophilicity of Ticks

Based on literature reports [54], the degree of ornithophilicity of the tick species was determined using the following scale: + accidentally ornithophilic, ++ relatively ornithophilic, +++ strictly ornithophilic.

#### 2.4.2. Quantitative Characteristics of the Population of Captured Birds

The population of the captured birds was characterized using the ecological index of dominance (DBs%) [49,50].

$$DBs=\frac{Bs}{Bt}\times 100\%$$

where *DBs*—ecological index of bird dominance, *Bs*—number of birds of a given species, *Bt*—total number of birds

The prevalence of tick infestation in a particular bird species (PBs%) and the mean intensity of tick infestation in a given bird species (MIIB) were calculated as follows [49,50,53].

$$\mathrm{PBs}=\frac{BsT}{Bs}\times 100\%$$

where *PBs*—prevalence of infested birds in captured individuals, *BsT*—number of birds of a given species infested with ticks, *Bs*—number of captured birds of a given species

$$\mathrm{MIIB}=\frac{TBs}{BsT}$$

where *MIIB*—mean intensity of tick infestation in a given bird species, *TBs*—number of all tick species collected from a particular bird species, *BsT*—number of birds of a given species infested with all ticks

### 2.5. Behavior of Captured Birds

The captured birds were classified into ecological types based on the foraging style criterion proposed by Ciebiera et al. [36]: Type 0—birds foraging mainly on the ground and Type 1—birds foraging mainly in shrubs. Additionally, they were classified according to the distances covered during the seasonal migrations. Group 1 comprised sedentary/local migrants, i.e., birds staying in the same region throughout the season or migrating over short distances, usually not reaching the Mediterranean basin for overwintering. Group 2 included medium-distance migrants, usually overwintering in the Mediterranean Basin. Group 3 comprised long-distance migrants, i.e., those covering long distances between continents and usually overwintering in central or southern Africa. The birds were also characterized in terms of migration behavior: d—diurnal—daytime migration, n—nocturnal—night migration, and n/d—beginning of the flight at night and continuation during the day [36].

### 2.6. Statistical Analysis

The type of distribution of the analyzed data was checked using the Shapiro–Wilk test. Due to the lack of normality of the distribution, non-parametric tests were used for the statistical calculations.

The statistical significance of differences in the prevalence of tick infestation between the bird species was tested using the chi-square test. The significance of the differences in the mean intensity of tick infestation depending on birds’ behavior was analyzed using the Mann–Whitney U test. The relationships between birds’ body weight, amount of fat tissue, and tick infestation intensity were tested using Spearman’s rho correlation.

In all statistical tests, the results at *p* < 0.05 were considered significant. The statistical analysis was performed using GraphPad 8.1 software (GraphPad Software, San Diego, CA, USA).

## 3. Results

### 3.1. Prevalence of Tick Infestation in Migratory Birds

**Figure 2:**
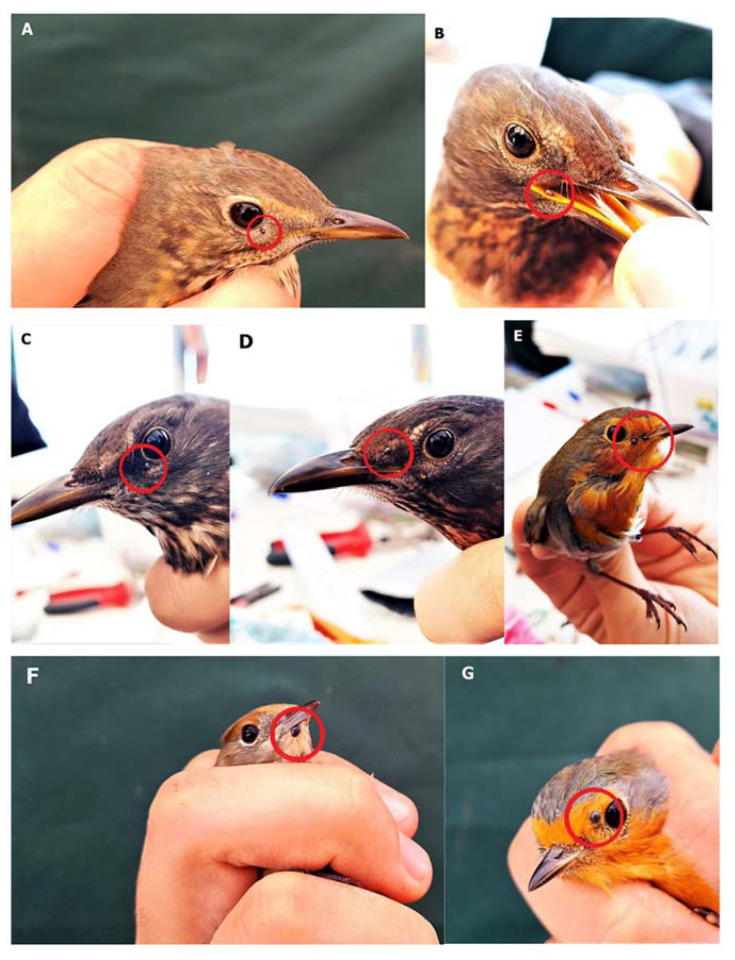
*Ixodes ricinus* ticks infesting birds. (**A**) *Turdus philomelos*, larva attached to the eyelid; (**B**) *T. merula*, nymph in the gape; (**C**) *T. merula*, nymph feeding near the eyelid; (**D**) *T. merula*, nymph feeding on the lore; (**E**) *Erithacus rubecula*, nymphs feeding on the lore; (**F**) *Sylvia atricapilla*, nymph attached to the chin; (**G**) *E. rubecula*; nymph feeding on the lore. Photo by Z. Zając.

In total, 3903 birds of 55 different species were captured during the field study. Among the captured bird species, *Erithacus rubecula* Linnaeus, 1758 (1142); *Sylvia atricapilla* Linnaeus, 1758 (638); *Turdus merula* Linnaeus, 1758 (495); and *T. philomelos* Brehm, 1831 (341) were the most frequent. Tick infestation was confirmed in only 11 of 55 captured bird species (Table 1 and Table S3). The most common locations for tick attachment were the eyelid and chin (Figure 2). The total prevalence of tick infestation was 4.43% (173 infested of 3903 captured birds), with a statistically significant difference in prevalence of tick infestation between individual bird species (χ^2^ = 46.12, *p* < 0.001).

### 3.2. Tick Species and Tick Parasitic Stages Infesting Migratory Birds

Seven species of *Ixodes* were identified among the 335 ticks collected from the birds. These included *I. ricinus*, *I. persulcatus* Schulze, 1930; *I. frontalis* Panzer, 1798; *I. trianguliceps* Birula, 1895; *I. crenulatus* Koch, 1849 syn. *I. canisuga*; *I. apronophorus* Schulze, 1924; *I. acuminatus* Neumann, 1901, syn. *I. redikorzevi*. No ticks from the other genera were found (Table 2).

**Figure 3:**
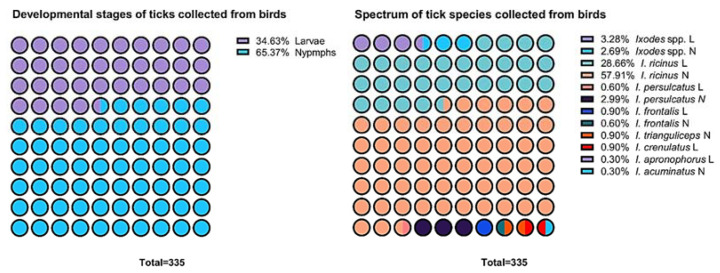
Quantitative and qualitative structure of collected ticks. Nymphs are only the developmental stages of ticks collected from birds. *I. ricinus* is the most frequent tick species infesting birds.

Only juvenile stages of ticks with a predominance of nymphs (65.37%) over larvae (34.63%) were found in the entire study group (Figure 3). The ticks collected from the captured birds were characterized by considerable ecological dominance (DTs) of *I. ricinus* (86.56%) (Table 2 and Table 3). Moreover, the highest mean infestation intensity (MII) was determined for this tick species (Table 3). *I. ricinus* ticks were collected from 9 bird species, accounting for 80.12% of all birds captured in the nets (Table 2 and Table 3). In contrast, *I. crenulatus*, *I. apronophorus*, and *I. acuminatus* were found in only one bird species each (Table 2).

The bird species differed in the number of infesting tick species. The greatest number of different species of ticks was collected from *E. rubecula* (5 out of the 7 identified species of ticks) (Table 2). Only one of the tick species collected from the birds (i.e., *I. frontalis*) was strictly ornithophilic (Table 3).

### 3.3. Intensity of Tick Infestation Depending on Bird Behavior

**Figure 4:**
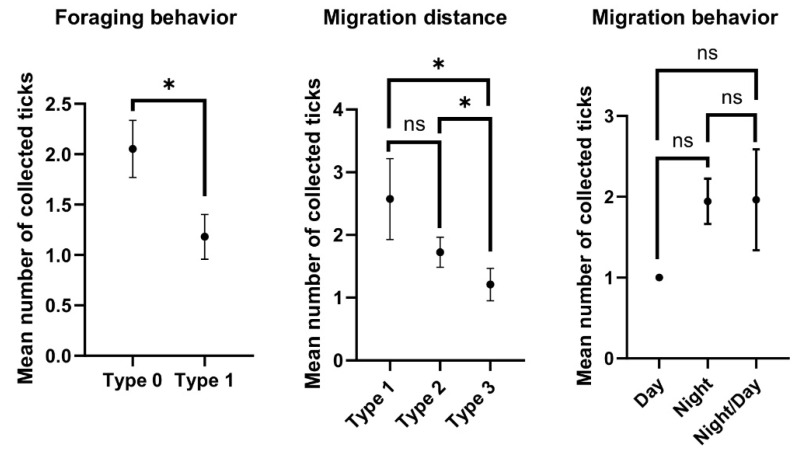
Mean intensity of tick infestation depending on the birds’ behavior (including the 95% confidence interval), *—statistically significant differences, ns—statistically insignificant differences.

The highest mean infestation intensity (2.05 ticks/bird) was observed in the ground or low plant feeding bird species (type 0), whereas the value of this parameter in the group of birds feeding mainly in shrubs was 1.18 tick/bird (type 1); these differences were statistically significant (*p* = 0.0047) (Figure 4). Significant differences in the intensity of tick infestation were also observed between sedentary/local migrant birds (type 1) and long-distance migrants (type 3) (*p* = 0.0058) and between medium-distance migrants (type 2) and long-distance migrants (type 3) (*p* = 0.0425). In turn, the differences in the intensity of tick infestation between species exhibiting diurnal (1.0 tick/bird), nocturnal (1.94 tick/bird), and nocturnal/diurnal (1.96 tick/bird) migration behavior were not statistically significant (Figure 4).

### 3.4. Tick Host Preferences

The collected tick species differed in terms of the host preference index (*I_p_*). The strongest tick–host relationship was observed between *T. philomelos*, *T. merula*, *E. rubecula,* and ticks. In turn, the most frequent tick species collected from birds, i.e., *I. ricinus*, exhibited a preference for the infestation of *T. merula* (3.42). The lowest values were recorded for the *P. major* and *I. ricinus* pairs (0.06) (Table 4).

A weak negative but statistically significant correlation was observed between the bird’s body weight and the prevalence of tick infestation only in *E. rubecula* (r_s_ = −0.2563, *p* = 0.0265). There was no significant correlation between the amount of fat tissue and tick infestation in any bird species.

## 4. Discussion

Migratory birds play a significant role in introducing new tick species to areas previously considered tick-free [12,13,14,45], thereby posing a risk of local outbreaks of tick-borne diseases [55,56]. The results of the present study show that *I. ricinus* is the most frequently collected tick species from birds during their autumn migration (86.56% of all collected ticks) in eastern Poland. It is also the most widespread tick species in Europe [45], and its dominance on migratory birds has been reported in other studies conducted on the continent [7,15,18,23,25,35,36]. In the present study, we identified tick species that, to the best of our knowledge, had not been reported from eastern Poland (*I. persulcatus*, *I. frontalis*, *I. acuminatus*) or had been reported only sporadically (*I. trianguliceps*, *I. crenulatus*, *I. apronophorus*) [46,57,58]. Among the ticks identified in the current study, *I. frontalis* and *I. ricinus* are the most frequent tick species collected from birds [38,46]. *I. persulcatus* specimens parasitizing birds were confirmed, among others, by studies in Sweden, on birds captured at their stopover sites in Yakutia (Asia) and even from migrating birds in Japan [59,60,61]. In the case of *I. acuminatus,* the main spectrum of hosts is small mammals of the genera *Microtus*, *Sorex*, and *Apodemus* [46,62], while birds are considered exceptional hosts for this species [48]. In turn, Filippova [63] considered birds to be important hosts, mainly for the *I. acuminatus* larvae. These observations are also confirmed by the results of studies from Romania [64]. In turn, *I. trianguliceps* is a hydrophilic tick species that occurs in wet mixed forest habitats. This tick species is mostly found near the burrows of potential hosts, mainly small mammals [47,65]. Such behavior promotes ticks to attack birds that usually forage on the ground or in low parts of plants (foraging type 0 and/or 1) (Table 2). Feeding of this tick species on birds has also been confirmed [46,47]. Similar host preferences are observed in *I. crenulatus* and *I. apronophorus* occurring in river valleys, which are habitats of birds [47].

Particularly noteworthy is the relatively large number of collected larvae (2) and nymphs (10) of *I. persulcatus* (Table 2), whose compact range covers northeastern Europe [66,67]. In the present study, this species was collected from short-distance (*T. merula*) and medium-distance (*E. rubecula*, *T. philomelos*) migrants (Table 2), who migrated from the north and northeast of the continent to the Mediterranean region in autumn. Considering the feeding time of juvenile stages of ixodid ticks [68] and the distance covered daily by, e.g., *E. rubecula* [41], it can be suggested that these birds may play a significant role in the introduction of ticks in the eastern Poland region. However, our earlier study on the occurrence of ticks in this region did not confirm the presence of *I. persulcatus* [27,28]. Due to the northern range of its occurrence and the progressive climate change, this tick species may not find favorable habitat conditions in eastern Poland. Another noteworthy finding is the absence of *D. reticulatus*, i.e., the dominant tick species in eastern Poland [27,69], on the birds examined in this study. This is most probably related to the host specificity of these ticks. *D. reticulatus* larvae and nymphs typically infest small rodents [70] and are only sporadically found on birds [56,71].

In contrast to the results reported by other authors [4,72], we identified the presence of only *Ixodes* ticks on the birds (Table 2, Figure 3). This is probably associated with the location of the region along the birds’ autumn migration route from the north and north-east (dominated by *I. ricinus* and, to a lesser extent, by *I. persulcatus*) [73] to the south of the continent [41]. In Central Europe, in addition to the dominant *Ixodes* ticks, birds are most frequently infested by *H. marginatum* Koch, 1844; *Haemaphysalis punctata* Canestrini and Fanzago, 1878; *Hae. concinna* Koch, 1844; and less often by *D. reticulatus* [22,36,74]. 

The present study demonstrated the presence of ticks in 11 of the 55 identified bird species (20.00%), and the individuals representing the tick-infested species accounted for 82.09% (3204) of all captured birds (Table 1 and Table S3). Many tick-free bird species were captured in small numbers, excluding any reasoning about the lack of infestation. However, in this group there were also some species represented by a considerable number of individuals, and most of them were the ones foraging mainly in the tree canopy or higher bushes where ticks do not occur, e.g., *Cyanistes caeruleus* Linnaeus, 1758; *Regulus regulus* Linnaeus, 1758; *Muscicapa striata* Pallas, 1764; *Periparus ater* Linnaeus, 1758; or *Fringilla coelebs* Linnaeus, 1758 (Table S3). The presence of ticks in 61.11% of captured bird species was reported in studies conducted in west-central Poland, whereas tick infestation was observed in 55.55% of birds captured on the Baltic Sea coast [36,37]. These findings confirm the important role of birds as tick hosts and the involvement of birds in the spread of ticks. Although birds are not considered primary ticks’ hosts and the main reservoir of tick-borne pathogens in the environment [70,75], the broad spectrum of infested species increases the risk of transmission of pathogens; hence, birds should be considered an important link in the circulation of tick-borne pathogens.

In the present study, 4.43% of all captured birds were infested by ticks (Table 1). The lower prevalence of tick infestation than that in other regions of Poland [36,37] is probably associated with the fact that the study was conducted only in autumn (limitation related to coordination with ornithological station dates), while tick infestation rates are usually higher in spring [46,59,76].

The results of the present study indicate the dominance of nymphs (65.37%) over larvae (34.63%) infesting birds (Figure 3) in contrast to findings reported by other authors indicating the predominance of larvae in autumn [36,77,78]. In our opinion, these differences may be related to fluctuations in the seasonal activity of ticks.

The highest ecological index of dominance, the highest mean intensity of tick infestation, and a high host preference index were determined mainly in the case of short- and medium-distance migrants and species foraging on the ground or in low shrubs, i.e., *T. merula*, *T. philomelos*, *E. rubecula*, and *S. atricapilla* (Table 1, Table 2 and Table 4; Figure 4). Similar dependencies between the tick infestation levels and the distance covered by birds during seasonal migrations were also observed in, e.g., Scandinavia [77,79]. The birds’ foraging style should be regarded as an important factor influencing tick infestation intensity, especially bearing in mind that most of the tick species collected in this study are classified as relatively ornithophilic, including *I. ricinus* [54] (Table 3). Birds foraging on the ground are much more likely to be attacked by juvenile tick stages than those foraging in shrubs (Table 2). The present study showed ticks attached only to the heads of the birds. The most common location was the region around the eye and beak (Figure 2), which had direct contact with the ground during foraging. Moreover, the head is a safe place for the tick to attach, as it cannot be groomed via the beak or claws [80]. 

Our results also showed a significant (but weak) negative correlation between bird’s body weight and tick infestation intensity in the case of *E. rubecula*. This relationship may have been related to the fact that parasite infection increases hosts’ stress and may lead to a decrease in body mass in strongly infested individuals [1]. In addition, the immune status of the host may also influence the intensity of tick infestation in birds [81]. In addition, there are probably no genetic resistance mechanisms contributing to tick infestation in birds [81,82]. 

## 5. Conclusions

In conclusion, *I. ricinus* is the most common tick species infesting the examined bird species during their autumnal migration in eastern Poland. Similarly, captured Passeriformes species, such as *T. merula*, *T. philomelos*, *E. rubecula*, and *S. atricapilla*, should be regarded as the preferred tick hosts. They play an important role in the spread of rare tick species in this region. Avian ecology and ethology, foraging styles, and length of migration distance have an impact on the exposure of birds to tick attacks and indirectly contribute to the maintenance and circulation of tick-borne pathogens in the environment. 

## Figures and Tables

**Table 1:** Tick infestation among the analyzed bird species.

Bird Species	Bs	DBs (%)	BsT	TBs	PBs (%)	MIIB
*Acrocephalus schoenobaenus* Linnaeus, 1758	6	0.15	1	1	16.67	1.00
*Erithacus rubecula* Linnaeus, 1758	1142	29.26	77	122	6.74	1.58
*Parus major* Linnaeus, 1758	241	6.17	2	2	0.83	1.00
*Phylloscopus collybita* Vieillot, 1817	148	3.79	1	1	0.67	1.00
*Poecile montanus* Conrad, 1827	13	0.33	1	1	7.69	1.00
*Sylvia atricapilla* Linnaeus, 1758	638	16.35	13	19	2.04	1.46
*Sylvia borin* Boddaert, 1783	64	1.64	1	1	1.56	1.00
*Sylvia communis* Latham, 1787	10	0.26	1	1	10.00	1.00
*Troglodytes troglodytes* Linnaeus, 1758	106	2.72	2	2	1.89	1.00
*Turdus merula* Linnaeus, 1758	495	12.68	49	134	9.90	2.73
*Turdus philomelos* Brehm, 1831	341	8.74	25	51	7.33	2.04

Bs—number of captured birds of particular species, DBs—index of ecological dominance of bird species, BsT—number of birds of particular species infested with ticks, TBs—number of ticks collected from a particular bird species, PBs—prevalence of tick infestation among particular bird species, MIIB—mean intensity of tick infestation of a particular bird species.

**Table 2:** Ticks infesting birds and bird behavior type.

Bird Species	Number of Captured Birds	Bird Behavior Type			Tick Species																										
					*Ixodes* spp.			*Ixodes ricinus* Linnaeus, 1758			*Ixodes persulcatus* Schulze, 1930			*Ixodes frontalis* Panzer, 1798			*Ixodes trianguliceps* Birula, 1895			*Ixodes crenulatus* Koch, 1849 syn. *I. canisuga*			*Ixodes apronophorus* Schulze, 1924			*Ixodes acuminatus* Neumann, 1901 syn. *I. redikorzevi*			Total		
		FT	MD	MB	L	N	T	L	N	T	L	N	T	L	N	T	L	N	T	L	N	T	L	N	T	L	N	T	L	N	T
*Acrocephalus schoenobaenus* Linnaeus, 1758	6	1	3	n	-	-	-	-	1	1	-	-	-	-	-	-	-	-	-	-	-	-	-	-	-	-	-	-	0	1	1
*Erithacus rubecula* Linnaeus, 1758	1142	0	2	n	9	6	15	35	62	97	1	2	3	3	-	3	-	-	-	3	-	3	1	-	1	-	-	-	52	70	122
*Parus major* Linnaeus, 1758	241	1	1	d	1		1	1	-	1	-	-	-	-	-	-	-	-	-	-	-	-	-	-	-	-	-	-	1	1	2
*Phylloscopus collybita* Vieillot, 1817	148	1	3	n/d	-	-	-	-	1	1	-	-	-	-	-	-	-	-	-	-	-	-	-	-	-	-	-	-	0	1	1
*Poecile montanus* Conrad, 1827	13	1	1	d	-	-	-	-	-	-	1	-	1	-	-	-	-	-	-	-	-	-	-	-	-	-	-	-	1	1	1
*Sylvia atricapilla* Linnaeus, 1758	638	1	3	n	-	-	-	4	14	18	-	-	-	-	-	-	-	1	1	-	-	-	-	-	-	-	-	-	4	15	19
*Sylvia borin* Boddaert, 1783	64	1	3	n	-	-	-	-	-	-	-	-	-	-	-	-	-	-	-	-	-	-	-	-	-	-	1	1	0	1	1
*Sylvia communis* Latham, 1787	10	1	3	n	-	-	-	-	1	1	-	-	-	-	-	-	-	-	-	-	-	-	-	-	-	-	-	-	0	1	1
*Troglodytes troglodytes* Linnaeus, 1758	106	0	1	n	-	-	-	1	1	2	-	-	-	-	-	-	-	-	-	-	-	-	-	-	-	-	-	-	1	1	2
*Turdus merula* Linnaeus, 1758	495	0	1	n	1	2	3	38	87	125	-	4	4	-	2	2	-	-	-	-	-	-	-	-	-	-	-	-	39	95	134
*Turdus philomelos* Brehm, 1831	341	0	2	n/d	-	1	1	17	27	44	-	4	4	-	-	-	-	2	2	-	-	-	-	-	-	-	-	-	17	34	51
Total	3204				11	9	20	96	194	290	2	10	12	3	2	5	0	3	3	3	0	3	1	0	1	0	1	1	116	219	335

FT—foraging type, 0—birds foraging mainly on the ground, 1—birds foraging mainly in shrubs; MD—migration distance, 1—sedentary/local migrants, 2—medium-distance migrants, 3—long-distance migrants; MB—migration behavior, d—diurnal, n—nocturnal; n/d—nocturno-diurnal, L—larva, N—nymph, T—total number. When no tick was collected from a particular bird species, the record was marked with “-”.

**Table 3:** Quantitative characteristics of ticks collected from captured birds.

Tick Species	Ornithophilic Scale	DTs (%)	PTs(%)	NHSPI	MII
*Ixodes* spp.		5.97	0.41	5	1.25
*Ixodes ricinus* Linnaeus, 1758	++	86.56	3.77	9	1.97
*Ixodes persulcatus* Schulze, 1930	+	3.58	0.31	4	1.00
*Ixodes frontalis* Panzer, 1798	+++	1.49	0.10	2	1.25
*Ixodes trianguliceps* Birula, 1895	+	0.90	0.05	2	1.50
*Ixodes crenulatus* Koch, 1849 syn. *I. canisuga*	+	0.90	0.03	1	3.00
*Ixodes apronophorus* Schulze, 1924	+	0.30	0.03	1	1.00
*Ixodes acuminatus* Neumann, 1901 syn. *I. redikorzevi*	+	0.30	0.03	1	1.00

DTs—ecological index of tick species dominance; PTs—prevalence of particular tick species in the captured birds; NHSPI—number of host species infested with particular tick species; MII—mean intensity of infestation by tick species; + accidentally ornithophilic, ++ relatively ornithophilic, +++ strictly ornithophilic.

**Table 4:** Index of host preferences of ticks collected from the birds (only bird species with at least 100 captured individuals were included in the analysis).

Bird Species	Tick Species						
	*Ixodes* spp.	*Ixodes ricinus* Linnaeus, 1758	*Ixodes persulcatus* Schulze, 1930	*Ixodes frontalis* Panzer, 1798	*Ixodes trianguliceps* Birula, 1895	*Ixodes crenulatus* Koch, 1849 syn. *I. canisuga*	*Ixodes apronophorus* Schulze, 1924
*Erithacus rubecula* Linnaeus, 1758	2.56	1.14	0.85	2.05	-	3.42	3.42
*Parus major* Linnaeus, 1758	0.81	0.06	-	-	-	-	-
*Phylloscopus collybita* Vieillot, 1817	-	0.09	-	-	-	-	-
*Sylvia atricapilla* Linnaeus, 1758	-	0.38	-	-	2.04	-	-
*Troglodytes troglodytes* Linnaeus, 1758	-	0.13	-	-	-	-	-
*Turdus merula* Linnaeus, 1758	1.18	3.42	2.63	3.15	-	-	-
*Turdus philomelos* Brehm, 1831	0.57	1.74	3.82	-	7.63	-	-

*I_p_* < 1—negative interaction; *I_p_* = 1—neutral interaction; *I_p_* > 1—positive interaction. When no calculation was performed due to the absence of particular tick species on particular bird species, the record was marked with “-”.

## Data Availability

All data generated in this study have been published in this article.

## Supplementary Materials

The following supporting information can be downloaded at: https://www.mdpi.com/article/10.3390/ijerph192113781/s1, Table S1. Morphological characteristics of larvae belonging to tick species identified in the current study; Table S2. Morphological characteristics of nymph belonging to tick species identified in the current study; Table S3. Bird species captured during the current study.
