# Supplementary material for: Tick Infestation in Migratory Birds of the Vistula River Valley, Poland
Source: Int J Environ Res Public Health. 2022 Oct 23;19(21):13781. doi: 10.3390/ijerph192113781 (PMC9655835; doi:10.3390/ijerph192113781)
Supplement: Supplementary file 1 [file ijerph-19-13781-s001.zip › Table S1.pdf]

Table S1. Morphological characteristics of larvae belonging to tick species identified in the current study. Based on Estrada-Peña et al. [45], Siuda [46] and Nowak-Chmura [47].

**Body parts**	***I. ricinus***	***I. persulcatus***	***I. frontalis***	***I. crenulatus* syn. *I. canisuga***	***I. apronophorus***
Idiosoma	Scutum wider than longer. Short scutal setae. Four pairs of medial dorsal setae, six pairs of posterolateral setae.	Scutum wider than longer. Scutum the widest in the midle of its lenght. Three pairs of medial dorsal setae. Intercoxal setae not split.	Oval scutum wider than longer. Scutum the widest in the middle of its lenght.	Scutum long, heart-shaped, tapered in posterior half and the widest in the 1/3 length of frontal region. Seven pairs of marginal dorsal setae. Five pairs of of scutal setae. Intercoxal setae of equal lenght.	Pentagonal scutum, wider than longer. Posterolateral edge of scutum is concaved while lateral edge – widely rounded. Four pairs of medial dorsal setae. Six pairs of posterolateral setae. Intercoxal setae with split endings.
Gnathosoma	Basis capituli with dorsal auriculae. Indistinctive II and III palpal segments. Hypostome with dentition 3/3. Large auriculae.	Dorsal basis capituli with distinctive horns with rounded apex. Distinctive II and III palpal segments. Hypostome with dentition 3/3. Triangular auriculae.	Basis capituli of trapezoidal shape with no dorsal auriculae. Club-like palps. Segment I of palps partially fused with basis capituli. Boundary between II and III segments invisible. Hypostome with dentition 3/3.	Basis capituli with no dorsal horns and auriculae. Club-like palps. Boundary between II and III segments unclear. Palps with waved external edges. Hypostome the widest in frontal 1/3 of its lenght, dentition 4/4.	Triangular dorsal side of basis capituli with narrow dorsal horns. Large auriculae on ventral basis capituli. Strait basis capituli with small concave. Palps of equal width on its whole lenght. Boundary between II and III palpal segments unclear. Palps the widest in the half of the III segment. Hypostome with dentition 3/3.
Legs	Posteromedian spur on coxa I. Posterolateral spur on coxa I and II. Coxa II and III with outgrowths.	Coxa I with posteromedian spur. Coxa I-III with triangular, posterolateral spur.	Coxa I with two posterior spurs of the same size. Coxa II and III with posterolateral spur, bigger than posterior spur on coxa I.	Coxa I with wide but short median spur. All coxa with no posterolateral spur. Coxa III clearly larger than coxa I.	Coxa I with posteromedian spur. Shorter posterolateral spur present. Coxa II and III with outgrowths.
