# Supplementary material for: Tick Infestation in Migratory Birds of the Vistula River Valley, Poland
Source: Int J Environ Res Public Health. 2022 Oct 23;19(21):13781. doi: 10.3390/ijerph192113781 (PMC9655835; doi:10.3390/ijerph192113781)
Supplement: Supplementary file 1 [file ijerph-19-13781-s001.zip › Table S2.pdf]

Table S2. Morphological characteristics of nymph belonging to tick species identified in the current study. Based on Estrada-Peña et al. [45], Siuda [46] and Nowak-Chmura [47].

**Body parts**	***I. ricinus***	***I. persulcatus***	***I. frontalis***	***I. trianguliceps***	***I. acuminatus* syn. *I. redikorzevi***
Idiosoma	Oval idiosoma. Pentagonal scutum. Genital slit not connected frontally. Round spiracular plates. Visible buds of gential opening. Scutal setae short and shorter than dorsal lateral setae.	Oval scutum, anteriorally narrowed. Genital groove not connected frontally, goes from IV pair of legs to posterior edge of idiosoma. Visible buds of gential opening. Scutal setae short with split endings. Lack of intercoxal setae between II pair of coxa.	Scutum irregularly pentagonal with rounded posterior edge. Anal groove of horse-shoe shape and do not reach to posterior edge of idiosoma. Medial scutal setae five times shorter than anterior setae. Five pairs of intercoxal setae. 6-7 pairs of ventral, posterolateral setae.	Pentagonal scutum, posteriorally rounded. Scutal setae present. Genital grooved formed by two straight lines going from II pair of coxa to posterior edge of idiosoma. Anal groove surrounds anal opening anteriorally. Two pairs of anal setae. Round spiracular plates.	Oval idiosoma. Numerous, long setae present on dorsal surface of idiosoma. Scutum elongated with arch-shaped posterior edge. Anterior scutal setae shorter than posterior. Diamter of spiracular plates ca. 1.5 times longer than diameter of anal ring.
Gnathosoma	Dorsal basis capituli with visible horns. Large auriculae. Distinctive palpal segments.	Dorsal basis capituli with large dorsal horns. Large and acute auriculae. Slender palps with distinctive II and III segments. Hypostome with dentition 3/3.	Dorsal basis capituli of irregular shape with constrictions and with small but distinctive dorsal horns. Auriculae present. Club-like palps. I segment of palps fused with basis capituli and indistinctive. Boundary between II and III segment of palps poorly visible. Hypostome with dentition 3/3.	Basis capituli of triangular shape, length of 0.26-0.30 mm. Palpal segment I wider than II. Distinctive II and III palpal segments. II palpal segment two times longer than III – triangular shaped. Hypostome lenght ca. 0.1 mm with dentition 2/2.	Dorsal basis capituli with triangular horns. Straight posterior edge of basis capituli. Convex shape of ventral posterior edge of basis capituli with no ventral horns. Auriculae of triangular shape. Palps equally wide on its whole lenght with visible concave lateral edge. Hypostome with dentition 2/2.
Legs	Coxa of I pair of triangular shape with posteromedian spur – the longest spurs present on coxa I and II.	Posteromedian spur only on coxa I. The largest posterolateral spur on coxa I and II, while the smallest on coxa IV.	Posteromedian, triangular spur present only on coxa I. Posterolateral spur present on coxa I-IV.	Legs shorter than in another *Ixodes* species. Rounded coxa with no posterior spurs. Prominent outgrowth on coxa I and II.	Coxa I-IV with posteromedian and posterolateral spurs. Posteromedian spur on coxa IV shorter than the rest of posterolateral spurs.
