# Supplementary material for: Tick Infestation in Migratory Birds of the Vistula River Valley, Poland
Source: Int J Environ Res Public Health. 2022 Oct 23;19(21):13781. doi: 10.3390/ijerph192113781 (PMC9655835; doi:10.3390/ijerph192113781)
Supplement: Supplementary file 1 [file ijerph-19-13781-s001.zip › Table S3.pdf]

Table S3. Bird species captured during the current study

Bird species infested with ticks	Number of captured birds	Bird species no infested with ticks	Number of captured birds
Acrocephalus schoenobaenus	6	*Accipiter nisus*	1
Erithacus rubecula	1142	*Acrocephalus palustris*	1
Parus major	241	*Acrocephalus scirpaceus*	1
Phylloscopus collybita	148	*Aegithalos caudatus*	23
Poecile montanus	13	*Aegolius funereus*	1
Sylvia atricapilla	638	*Alcedo atthis*	2
Sylvia borin	64	*Anthus trivialis*	3
Sylvia communis	10	*Caprimulgus europaeus*	1
Troglodytes troglodytes	106	*Certhia familiaris*	5
Turdus merula	495	*Chloris chloris*	9
Turdus philomelos	341	*Coccothraustes coccothraustes*	3
		Curruca communis	10
		Curruca curruca	7
		Cyanistes caeruleus	249
		Dendrocopos major	6
		Dendrocoptes medius	3
		Dryobates minor	4
		Emberiza citrinella	1
		Emberiza schoeniclus	1
		Ficedula hypoleuca	36
		Ficedula parva	1
		Fringilla coelebs	41
		Garrulus glandarius	8
		Hippolais icterina	5
		Lanius collurio	1
		Locustella fluviatilis	1
		Luscinia luscinia	1
		Muscicapa striata	76
		Periparus ater	43
		Phoenicurus ochruros	2
		Phoenicurus phoenicurus	5
		Phylloscopus sibilatrix	5
		Phylloscopus trochilus	12
		Picus viridis	1
		Poecile palustris	5
		Prunella modularis	20
		Pyrrhula pyrrhula	5
		Regulus ignicapilla	1
		Regulus regulus	76
		Scolopax rusticola	2
		Sitta europaea	8
		Spinus spinus	1
		Turdus iliacus	1
		Turdus pilaris	11
